# Alantolactone Suppresses Proliferation and the Inflammatory Response in Human HaCaT Keratinocytes and Ameliorates Imiquimod-Induced Skin Lesions in a Psoriasis-Like Mouse Model

**DOI:** 10.3390/life11070616

**Published:** 2021-06-25

**Authors:** Wen-Ho Chuo, Yu-Tang Tung, Chao-Liang Wu, Nicole R. Bracci, Yu-Kang Chang, Hung-Yi Huang, Chi-Chien Lin

**Affiliations:** 1 Department of Pharmacy, Tajen University, Pingtung 907, Taiwan; cwh@tajen.edu.tw; 2 Graduate Institute of Biotechnology, National Chung Hsing University, Taichung 402, Taiwan; peggytung@nchu.edu.tw; 3 Department of Medical Research, Ditmanson Medical Foundation Chia-Yi Christian Hospital, Chiayi City 600, Taiwan; wumolbio@mail.ncku.edu.tw; 4 Department of Biomedical Sciences and Pathobiology, Virginia-Maryland College of Veterinary Medicine, Virginia Polytechnic Institute and State University, Blacksburg, VA 24061, USA; Nbracci@vt.edu; 5 Department of Medical Research, Tungs’ Taichung Metro Harbor Hospital, Taichung 433, Taiwan; yogurt8306@gmail.com; 6 Department of Nursing, Jen-Teh Junior College of Medicine and Management, Miaoli 356, Taiwan; 7 Department of Dermatology, Ditmanson Medical Foundation Chia-Yi Christian Hospital, Chiayi City 600, Taiwan; 8 Department of Medical Research, China Medical University Hospital, Taichung 404, Taiwan; 9 Department of Medical Research, Taichung Veterans General Hospital, Taichung 407, Taiwan; 10 Department of Pharmacology, College of Medicine, Kaohsiung Medical University, Kaohsiung 807, Taiwan; 11 Institute of Biomedical Science, The iEGG and Animal Biotechnology Center, National Chung-Hsing University, Taichung 402, Taiwan

**Keywords:** alantolactone, cytokine, keratinocytes, STAT3, NF-κB p65, psoriasis

## Abstract

Psoriasis is an immune-mediated inflammatory disease that affects 2% to 3% of the world population. Alantolactone, a sesquiterpene lactone, was isolated from *Inula helenium* and *Radix inulae* and has several biological effects, including antifungal, anthelmintic, antimicrobial, anti-inflammatory, antitrypanosomal, and anticancer properties. This study aimed to evaluate the antipsoriatic potential of alantolactone in vitro and in vivo and to explore its underlying mechanisms. These results showed that alantolactone significantly attenuated IL-17A, IL-22, oncostatin M, IL-1α, and TNF-α (M5) cytokine-induced hyperproliferation in HaCaT keratinocytes. Moreover, M5 cytokines significantly upregulated the mRNA levels of TNF-α, IL-6, IL-1β, and IL-8. However, alantolactone attenuated the upregulation of these inflammatory cytokines. In addition, alantolactone was found to inhibit STAT3 phosphorylation and NF-κB p65 nuclear translocation in HaCaT keratinocytes. Furthermore, alantolactone treatment in mice significantly alleviated the severity of skin lesions (erythema, scaling and epidermal thickness, and inflammatory cell infiltration) and decreased the mRNA expression of inflammatory cytokines (e.g., TNF-α, IL-6, IL-1β, IL-8, IL-17A, and IL-23) in an IMQ-induced-like mouse model. Therefore, our new findings revealed that alantolactone alleviates psoriatic skin lesions by inhibiting inflammation, making it an attractive candidate for future development as an antipsoriatic agent.

## 1. Introduction

Psoriasis is a common chronic inflammatory skin disease that affects 2% to 3% of the world population [1]. Psoriasis is characterized by the excessive proliferation and abnormal differentiation of keratinocytes and excessive immune-cell infiltration in the dermis and epidermis [2]. The exact pathogenesis of psoriasis remains unclear, but it is a common immune-mediated disease believed to result from abnormal crosstalk between keratinocytes and immune cells [3]. Various cytokines secreted by immune cells stimulate keratinocytes, which may lead to keratinocyte hyperproliferation. Hyperproliferative keratinocytes respond to these cytokines by producing massive amounts of proinflammatory cytokines, thereby sustaining or even amplifying the inflammatory response [4,5,6,7]. Therefore, reducing keratinocyte hyperproliferation and/or excessive inflammation is useful for the treatment of psoriasis.

Alantolactone, a sesquiterpene lactone, is isolated from *Inula helenium* and *Radix inulae* and is commonly used in folk medicine [8]. Alantolactone has been reported to have a wide spectrum of biological effects, including antifungal, anthelmintic [9,10], antimicrobial [11,12], anti-inflammatory [13,14], and antitrypanosomal activities [13]. It also has antiproliferative effects on several cancer cell lines, such as colon, melanoma, ovarian, prostate, lung, and leukemia cell lines [15,16]. Wang et al. [17] showed that alantolactone alleviates inflammation, oxidative stress, and apoptosis pathways in a traumatic brain injury rat model. Dang et al. [18] pointed out that alantolactone suppresses inflammation, apoptosis, and oxidative stress through the activation of Nrf2/HO-1 and the inhibition of the NF-κB pathways of human bronchial epithelial cells induced with cigarette smoke. A previous study revealed that alantolactone could reduce the number of immune cells and decrease the expression and secretion of Th2 cytokines (IL-4 and IL-13) in the bronchoalveolar lavage fluid and lung tissues of ovalbumin-induced allergic asthmatic mice [19].

Mixed cytokines, including IL-17A, IL-22, oncostatin M, IL-1α, and TNF-α (M5), are used to simulate HaCaT keratinocytes in vitro to establish a psoriatic keratinocyte model that generates some common phenotypic features of psoriasis [20]. Therefore, HaCaT keratinocytes stimulated by M5 cytokines were used to establish a psoriasis keratinocyte model, which produces certain characteristics of common psoriasis in vitro [21,22,23]. The psoriasis model induced by imiquimod (IMQ) is very similar to human psoriasis and has been widely used to induce psoriasis-like mouse models [24]. In this study, our aim was to investigate the therapeutic effects of alantolactone on M5 cytokine-stimulated keratinocytes and IMQ-induced psoriasis-like dermatitis and to explore the underlying mechanism of action.

## 2. Material and Methods

### 2.1. Cell Viability Assay

Human keratinocyte HaCaT cells were obtained from the American Type Culture Collection (ATCC, Rockville, MD, USA) and cultured in DMEM supplemented with 10% FBS, 100 U/mL penicillin, and 100 μg/mL streptomycin. Cells were incubated in a humidified incubator (5% CO_2_ and 95% air) at 37 °C. HaCaT keratinocytes were seeded into a 96-well plate in triplicate and allowed to adhere overnight. Alantolactone (>98%, ChemFaces, Wuhan, China) was dissolved in dimethyl sulfoxide (DMSO, Sigma, St. Louis, MO, USA). First, 200 μL of fresh medium containing various concentrations of 0.1% DMSO (control) or alantolactone (1.25, 2.5, and 5 μM) was added to the cultures and incubated at 37 °C for 2 h prior to stimulation with M5 cytokines (IL-17A, IL-22, oncostatin M, IL-1α, and TNF-α, each at a final concentration of 2.5 ng/ml; the recombinant human proteins were all purchased from BioLegend) for an additional 72 h. After 72 h, a cell viability assay was performed with Cell Counting Kit-8 (CCK-8; Dojindo Molecular Technologies, Inc., Kumamoto, Japan) according to the manufacturer’s protocol. The cell viability ratio (%) was calculated from the following equation: % viability = (absorbance of test sample/absorbance of control) × 100%.

### 2.2. RNA Extraction and Quantitative Real-Time PCR Analysis

HaCaT keratinocytes were pretreated with 0.1% DMSO (control) or alantolactone (1.25, 2.5, and 5 μM) for 2 h prior to being stimulated with M5 cytokines for an additional 24 h. Skin tissues were collected on day 7. Total RNA was extracted from HaCaT cells or skin tissues using TRIzol reagent (Invitrogen; Thermo Fisher Scientific, Inc., Waltham, MA, USA). Total RNA was reverse-transcribed into cDNA using M-MLV reverse transcriptase (Promega Corporation, Madison, WI, USA). Real-time RT-PCR was performed using SYBR-Green PCR Master Mix (Applied Biosystems; Thermo Fisher Scientific, Inc.) in the ABI 7500 Fast Real-Time system (Applied Biosystems; Thermo Fisher Scientific, Inc.). To evaluate gene expression, real-time RT-PCR was performed on target genes (KRT6, TNF-α, IL-6, IL-1β, IL-8, IL-17A, and IL-23) using cDNA from HaCaT cells or skin tissues. The 2^−ΔΔCT^ method was used to determine the relative expression of target genes after normalization to glyceraldehyde 3-phosphate dehydrogenase (GAPDH). The primer pairs used are shown in Supplementary Table S1.

### 2.3. Western Blotting

HaCaT keratinocytes were pretreated with 0.1% DMSO (control) or alantolactone (1.25, 2.5, and 5 μM) for 2 h prior to being stimulated with M5 cytokines for an additional 30 min. Protein was extracted from HaCaT cells using RIPA buffer containing 1% protease inhibitor cocktail (Sigma-Aldrich, St. Louis, MO, USA). In addition, skin tissues were collected on day 7 and homogenized in 100 μL tissue RIPA lysis buffer for 30 min. Protein expression levels of pSTAT3, STAT3, and I-κBα were determined using Western blot analysis according to a method previously described by Li et al. [25]. In this study, the primary antibodies were anti-pSTAT3 (clone 13A31, BioLegend, San Diego, CA, USA), anti-STAT3 (clone 4G4B45, BioLegend), anti-I-κBα (clone 6A920, 169 Abcam, Cambridge, UK), and GAPDH (clone W17079A, BioLegend). Each membrane was reprobed with an antibody against GAPDH, which was used as an internal control for equal protein loading. The band density was quantified with ImageJ software (National Institute of Health, Bethesda, MD, USA).

### 2.4. Preparation of Nuclear Extracts and Measurement of NF-κB Activity

HaCaT keratinocytes were pretreated with 0.1% DMSO (control) or alantolactone (1.25, 2.5, and 5 μM) for 2 h prior to being stimulated with M5 cytokines for an additional 6 h. To evaluate NF-κB activity, cells from bone-marrow-derived dendritic cells (BMDCs) or skin tissues (on day 7) were used to obtain cytosolic and nuclear extracts using NE-PER (Cat# 78833, Thermo Fisher Scientific), a nuclear and cytoplasmic extraction reagent. The samples were used to measure NF-κB p65 subunit activation by a TransAM NF-κB p65 kit (Cat# 40098, Active Motif, Carlsbad, CA, USA) as instructed by each manufacturer’s manual and analyzed using a microplate reader at 450 nm, with a reference wavelength of 655 nm (Tecan Group Ltd., Männedorf, Switzerland).

### 2.5. Animals

Female BALB/c mice (7–8 weeks old) were provided by the National Laboratory Animal Center (Taipei, Taiwan) and were housed in a specific pathogen-free clean room under a 12 h light/12 h dark cycle. During the entire experiment, mice were given free access to food and water. Animal care was in accordance with the guidelines for the care and use of laboratory animals and the principles presented by the National Chung Hsing University, Taiwan (approval number NCHU-IACUC-110-024). All experimental procedures were conducted in strict accordance with the guidelines of the Animal Experiments Committee.

### 2.6. Preparation of Cream

The vehicle cream is composed of anhydrous lanolin and Vaseline at a ratio of 1:20. To prepare the alantolactone formulation, the vehicle cream was heated to 55 °C and mixed thoroughly with alantolactone (10 mg/mL (1%) or 20 mg/mL (2%)) by stirring.

### 2.7. IMQ-Induced Psoriasis-Like Mouse Model and Topical Alantolactone Treatment

The mice were randomly divided into 4 groups (n = 5): blank group, imiquimod (IMQ) (62.5 mg/day)-vehicle group, IMQ-1% alantolactone group, and IMQ-2% alantolactone group. Mice in the blank group did not receive any interventions; mice in the IMQ-vehicle group were treated with IMQ cream and vehicle cream (100 mg/day); mice in the alantolactone group were treated with IMQ cream, and 6 h later, were treated with 1% or 2% alantolactone formula (100 mg/day). The experimental period lasted for 6 consecutive days. During the entire experiment, the severity of back skin inflammation of the mice was scored individually regarding redness (erythema) and the presence of scales (scaling) on a scale from 0 to 4 (0, none; 1, slight; 2, moderate; 3, marked; 4, very marked) on days 1–7 by an independent investigator blinded to the groups. After the experiment, mice were euthanized by the inhalation of diethyl ether and cervical dislocation, and the back skin was immediately removed. The back skin tissue was fixed in 10% formalin and embedded in paraffin for histological analysis. The remaining skin tissue was stored at −80 °C for the extraction of RNA and total protein.

### 2.8. Histopathology and Immunohistochemistry

The paraffin-embedded (6 μm) sections were stained using hematoxylin and eosin (H&E) to study their microarchitecture. For immunohistochemical analyses, the sections were incubated overnight at 4 °C with primary rabbit antibodies against mouse CD3+ T cells (clone 17A2, Biolegend, San Diego, CA, USA) and Gr1+ neutrophils (RB6-8C5, Biolegend, San Diego, CA, USA) in PBS/0.1% BSA. Then, HRP-conjugated goat anti-rat secondary antibody was applied in PBS/0.1% BSA for 30 min at room temperature. Diaminobenzidine was used for staining development, and the sections were counterstained with hematoxylin. The negative control consisted of replacing the primary antibody with normal serum. Staining results were analyzed in a blind fashion by two independent investigators. For quantitative analysis, the integrated optical density (IOD) of Gr1+ and CD3+ cells was measured using ImageJ software (version 1.51n, Wayne Rasband, National Institutes of Health, USA).

### 2.9. Statistical Analysis

Experimental data are presented as the mean ± standard deviation (SD). Comparisons among multiple treatments were performed using Tukey’s HSD (honest significant difference) test after one-way ANOVA or two-way ANOVA by GraphPad Prism (version 8 for Windows; GraphPad Software, La Jolla, CA, USA). A *p* value < 0.05 was considered statistically significant.

## 3. Result

### 3.1. Alantolactone Inhibited Proliferation and Inflammatory Responses in M5 Cytokine-Stimulated Keratinocytes

**Figure 1:**
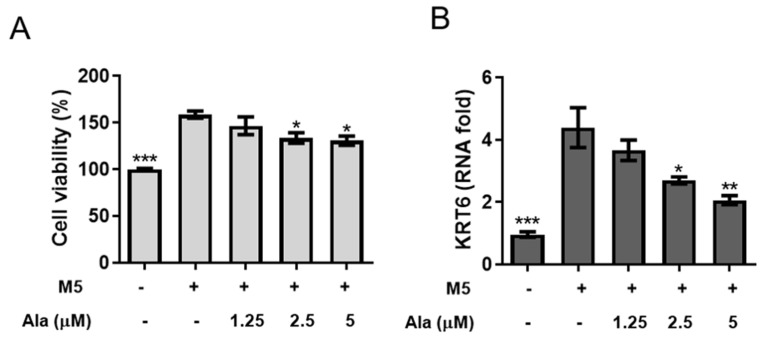
The effect of alantolactone on the proliferation of HaCaT keratinocytes stimulated with M5 cytokines. HaCaT keratinocytes were seeded in 96-well plates, treated with alantolactone (1.25, 2.5 and 5 μM) or 0.1% DMSO for 2 h, and subsequently stimulated with M5 cytokines (2.5 ng/mL) for 72 h. (**A**) CCK-8 was used to measure cell viability. (**B**) Cells were harvested, and RNA was extracted for qRT-PCR analysis of KRT6 mRNA levels. GAPDH served as an internal reference. The results of real-time PCR are presented as the fold change relative to the untreated control (set as 1.0). Values represent the means ± standard error of the mean (SEM) from triplicate samples for each treatment. (*) *p* < 0.05, (**) *p* < 0.01, (***) *p* < 0.001 versus the M5 + 0.1% DMSO-treated group, as determined by one-way ANOVA with Tukey’s multiple comparison test. Ala, alantolactone.

**Figure 2:**
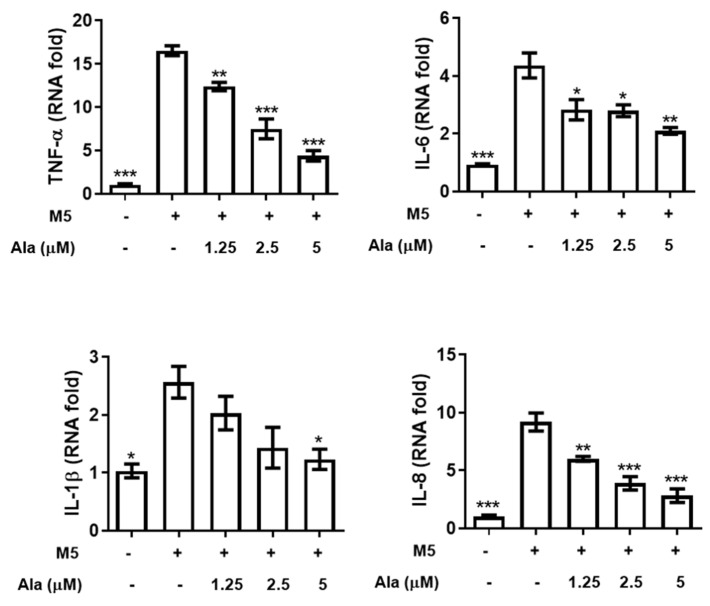
The effect of alantolactone on the inflammatory cytokine expression of HaCaT keratinocytes stimulated with M5 cytokines. HaCaT keratinocytes were seeded in 96-well plates and treated with alantolactone (Ala, 1.25, 2.5 and 5 μM) or 0.1% DMSO for 2 h and subsequently stimulated with M5 cytokines (2.5 ng/mL) for 24 h. Cells were harvested, and RNA was extracted for qRT-PCR analysis of TNF-α, IL-6, IL-1β, and IL-8 mRNA levels. GAPDH served as an internal reference. The results of real-time PCR are presented as the fold change relative to the untreated control (set as 1.0). Values represent the means ± SEM from triplicate samples for each treatment. (*) *p* < 0.05, (**) *p* < 0.01, (***) *p* < 0.001 versus the M5 + 0.1% DMSO-treated group, as determined by one-way ANOVA with Tukey’s multiple comparison test. Ala, alantolactone.

Mixed cytokines, including IL-17A, IL-22, oncostatin M, IL-1α, and TNF-α (M5), were used to simulate HaCaT keratinocytes to establish a psoriatic keratinocyte model that generates some phenotypic features in common psoriasis in vitro [18,19,20]. CCK-8 assays were used to investigate whether alantolactone affected the viability of M5 cytokine-stimulated HaCaT keratinocytes (Figure 1A). Figure 1A shows that 2.5 and 5 µM alantolactone significantly inhibited the proliferation of M5 cytokine-induced HaCaT cells at 72 h. Figure 1B shows that M5 cytokines significantly increased the mRNA levels of the hyperproliferative marker gene KRT6. Notably, alantolactone decreased KRT6 mRNA levels in a dose-dependent manner. In addition, M5 cytokines promoted the mRNA expression of inflammatory cytokines (TNF-α, IL-6, IL-1β, and IL-8) in HaCaT cells. However, the mRNA expression of these cytokines was significantly decreased by alantolactone (Figure 2). These results revealed that alantolactone suppressed M5 cytokine-induced proliferation and inflammatory responses in keratinocytes in vitro.

### 3.2. Alantolactone Inhibited STAT3 Phosphorylation and NF-κB Activation in HaCaT Keratinocytes

**Figure 3:**
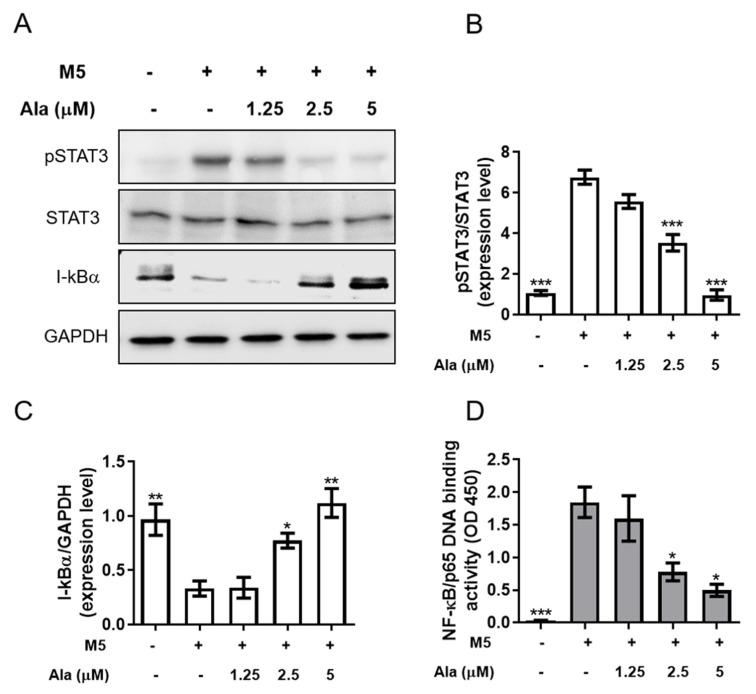
The effect of alantolactone on pSTAT3 and I-κBα expression and nuclear translocation of HaCaT stimulated with M5 cytokines. HaCaT keratinocytes were treated with alantolactone (Ala, 1.25, 2.5 and 5 μM) or 0.1% DMSO for 2 h and then stimulated with M5 cytokines (2.5 ng/mL) for 60 min. (**A**) Cells were harvested, and total protein was extracted for Western blotting to detect pSTAT3, STAT3, and I-κBα protein levels (Supplementary Figure S1). (**B**,**C**) GAPDH served as an internal reference, and the quantified expression levels by ImageJ software were plotted in the bar graphs. (**D**) NF-κB p65 DNA-binding activity in nuclear extracts of HaCaT cells was determined using the TransAM kit, representing the optical density at 450 nm (OD450). Values represent the means ± SEM from triplicate samples for each treatment. (*) *p* < 0.05, (**) *p* < 0.01, (***) *p* < 0.001 versus the M5 + 0.1% DMSO-treated group, as determined by one-way ANOVA with Tukey’s multiple comparison test. Ala, alantolactone.

STAT3 and NF-κB are activated in skin tissues in psoriasis [26]. In this study, M5 cytokines significantly induced STAT3 phosphorylation (Figure 3A,B) and decreased I-κBα (Figure 3A,C) expression, which could be recovered in the presence of alantolactone (Figure 3A–C). In unstimulated cells, an inactive latent form of p65 in the cytoplasm complexed with its inhibitor IκB-α. Figure 3D shows that 30 min of stimulation with M5 cytokines can induce robust translocation of p65 to the nucleus. However, p65 remained in the cytoplasm after alantolactone treatment.

### 3.3. Alantolactone Decreased the Severity of IMQ-Induced Psoriasiform Dermatitis

**Figure 4:**
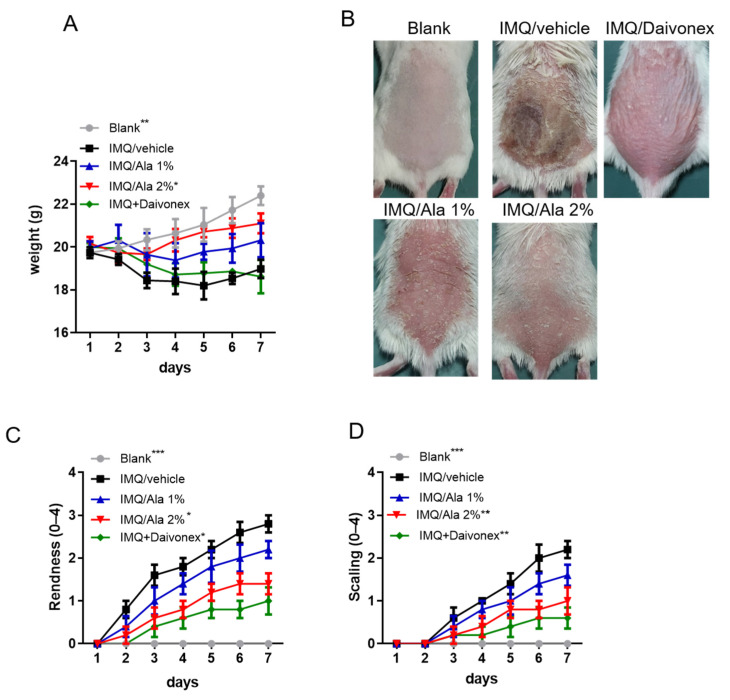
The effects of alantolactone on skin lesions of IMQ-induced psoriasis-like dermatitis in mice. (**A**,**B**) Daily mean disease severity is depicted as back skin redness and scaling scores for the mouse groups where Daivonex was used as a positive control. Symbols represent the mean score ± SEM of five mice per group. (**C**) Representative images of skin lesions in mice treated with IMQ or alantolactone. (**D**) Body weight changes are depicted for the mouse groups. The results are representative of three independent experiments. (*) *p* < 0.05, (**) *p* < 0.01, (***) *p* < 0.001 versus the IMQ/vehicle group, as determined by two-way ANOVA.

To evaluate the therapeutic efficacy of alantolactone treatment in an IMQ-induced psoriasis-like mouse model, mice were topically treated with alantolactone. Body weights of the IMQ-induced mice were significantly lower than those of the control group; however, alantolactone treatment allowed for an increase in the body weights of IMQ-induced mice (Figure 4A). Symptoms in the mice treated with alantolactone or Daivonex (a calcipotriol ointment; LEO Laboratories Limited) as a positive trial were noticeably milder than the mice treated with IMQ alone, with their back skin remaining smooth (Figure 4B). As shown in Figure 4C,D, IMQ-induced mice exhibited significant psoriasis-like skin that was characterized by redness and scaling of the skin lesion. However, alantolactone and Daivonex efficiently decreased the symptoms over the whole treatment period in a dose-dependent manner.

**Figure 5:**
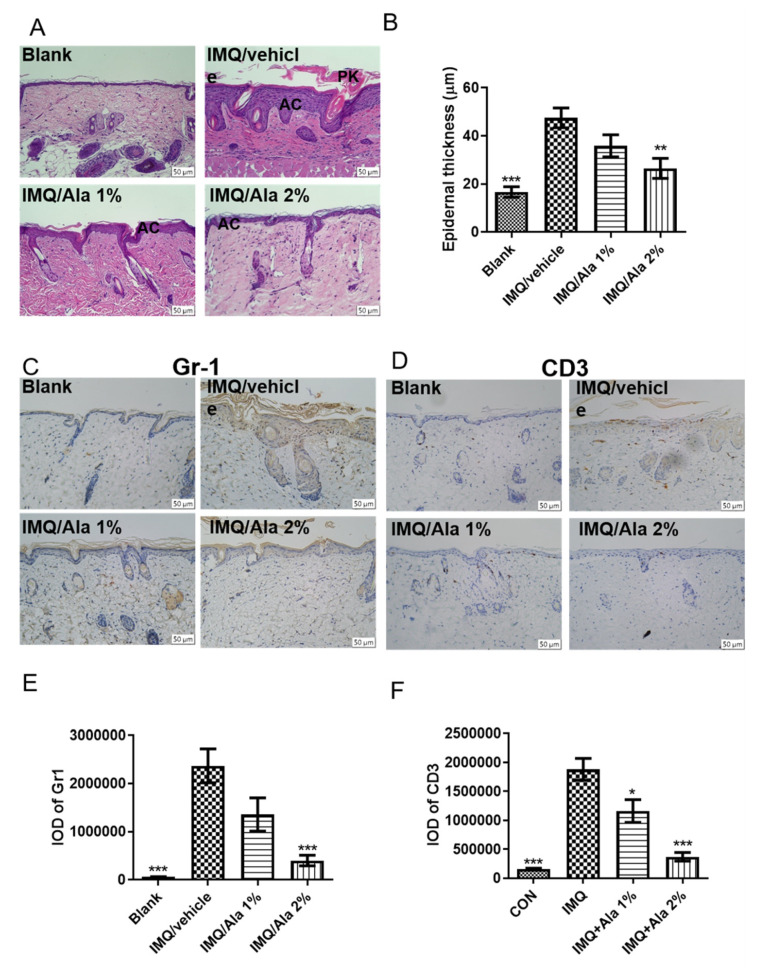
The effect of alantolactone on histological analysis of skin lesions of IMQ-induced psoriasis-like dermatitis in mice. (**A**) Representative H&E-stained back skin sections of different treatment groups. Acanthosis (AC), parakeratosis (PK). (**B**) Epidermal thickness of the dorsal skin on day 7. Representative staining for (**C**) GR1+ neutrophils. (**D**) CD3+ T-cells infiltrate the back skin of different mice groups on day 7. The integrated optical density (IOD) of (**E**) Gr1+ and (**F**) CD3+ T cells in the skin was calculated using ImagePro Plus. Each data point on the graph represents the mean ± SEM with five mice per group. The results are representative of three independent experiments. (*) *p* < 0.05, (**) *p* < 0.01, (***) *p* < 0.001 versus the IMQ/vehicle group, as determined by one-way ANOVA with Tukey’s multiple comparison test. Ala, alantolactone.

To further assess the therapeutic effects of alantolactone in IMQ-induced psoriasis-like dermatitis, the degree of skin inflammation on the back skin of the mice on day 7 after treatment was evaluated using histological (H&E) and immunohistochemical analyses. Figure 5 shows IMQ-induced epidermal thickness (acanthosis, AC) (Figure 5A,B), the presence of nuclei in the stratum corneum (parakeratosis, PK) (Figure 5A), increased level of neutrophils (GR-1+) (Figure 5C,D), and CD3+ T cell (Figure 5E,F) infiltration. However, topical treatment with alantolactone ameliorated these changes compared to those mice receiving IMQ alone. These results suggested that alantolactone could reduce IMQ-induced skin inflammation.

### 3.4. Alantolactone Decreased Inflammatory Cytokines in the Skin Lesions of IMQ-Treated Mice

**Figure 6:**
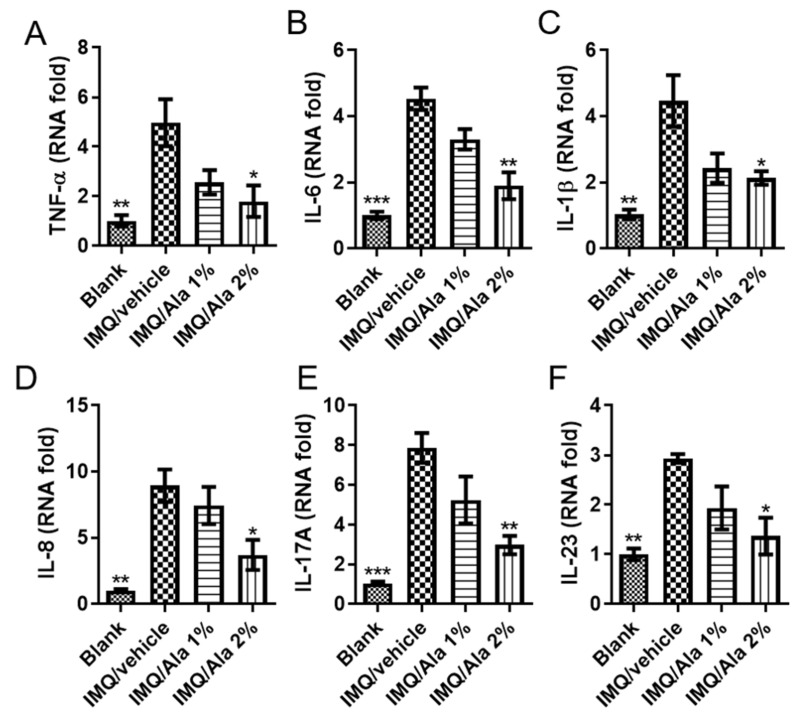
The effects of alantolactone on skin inflammatory cytokine production in the skin of IMQ-treated mice. Skin was excised from different treatment groups on day 7, and RNA was prepared to examine the mRNA expression of (**A**) TNF-α, (**B**) IL-6, (**C**) IL-1β, (**D**) IL-8, (**E**) IL-17A, and (**F**) IL-23 by quantitative RT-PCR. GAPDH served as an internal reference. The results of real-time PCR are presented as the fold change relative to untreated control mice (set as 1.0). Each data point on the graph represents the mean ± SEM with five mice per group. The results are representative of three independent experiments. (*) *p* < 0.05, (**) *p* < 0.01, (***) *p* < 0.001 versus the IMQ/vehicle group, as determined by one-way ANOVA with Tukey’s multiple comparison test. Ala, alantolactone.

To investigate the therapeutic efficacy of alantolactone on IMQ-induced dermatitis in mice, inflammatory cytokines within the skin of the mice were studied. As shown in Figure 6, the expression levels of all cytokines (TNF-α, IL-6, IL-1β, IL-8, IL-17A, and IL-23) in the IMQ-treated group were significantly higher than those in the control group. However, alantolactone treatment significantly reduced TNF-α, IL-6, IL-1β, IL-8, IL-17A, and IL-23 in skin lesions. Therefore, these results indicated that topical treatment with alantolactone resulted in a decrease in proinflammatory cytokines that are related to psoriasis pathogenesis in skin lesions following IMQ treatment.

### 3.5. Alantolactone Suppressed *STAT3 Phosphorylation and NF*-*κB Activation* in the Skin Lesions of IMQ-Treated Mice

**Figure 7:**
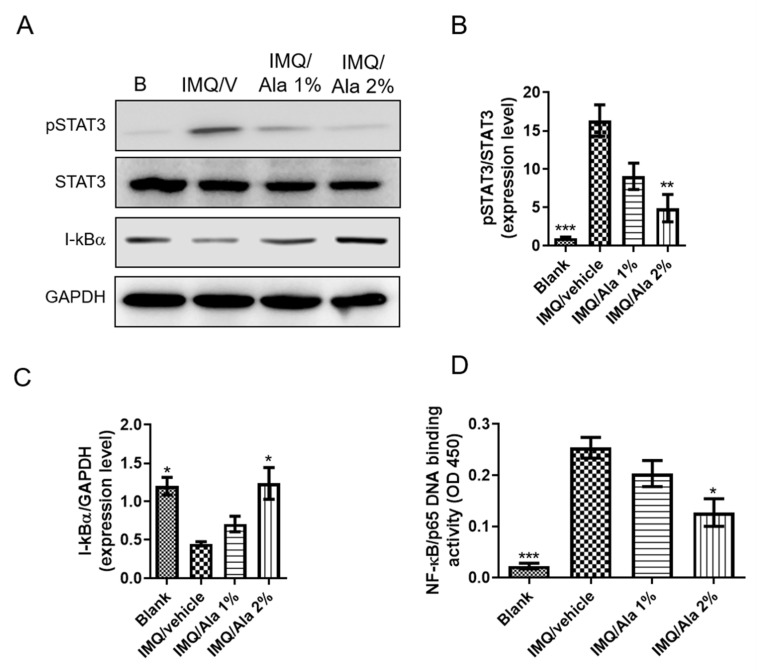
Effects of alantolactone on pSTAT3 and I-κBα expression and nuclear translocation in skin homogenates. Skin was excised from different treatment groups on day 7, and protein was prepared to examine protein expression. (**A**) The protein expression levels of pSTAT3, STAT3, and I-κBα were measured using Western blots (Supplementary Figure S2), and the quantified expressional levels of (**B**) pSTAT3/STAT3 ratio and (**C**) I-κBα with the internal reference, GAPDH, were analyzed with ImageJ software and plotted in the bar graphs using GraphPad software. (**D**) NF-κB p65 DNA-binding activity in nuclear extracts of skin homogenates was determined using the TransAM kit, representing the optical density at 450 nm (OD450). Each data point on the graph represents the mean ± SEM with five mice per group. The results are representative of three independent experiments. (*) *p* < 0.05, (**) *p* < 0.01, (***) *p* < 0.001 versus the IMQ/vehicle group, as determined by one-way ANOVA with Tukey’s multiple comparison test. B, Blank. IMQ/V, IMQ/vehicle. Ala, alantolactone.

To further assess the mechanism of alantolactone in IMQ-induced psoriasis-like dermatitis, STAT3 phosphorylation and NF-κB activation in affected skin were examined. As shown in Figure 7A,B, STAT3 phosphorylation was significantly increased in the skin of IMQ-induced mice. However, alantolactone significantly decreased STAT3 phosphorylation. In addition, to further investigate NF-κB activity, I-κBα and NF-κB p65 translocation was performed. As shown in Figure 7C,D, IMQ-induced mice significantly decreased I-κBα; however, alantolactone increased I-κBα expression and reduced NF-κB p65 subunit nuclear translocation in the skin tissues of IMQ-treated mice.

## 4. Discussion

Psoriasis is a complex inflammatory skin disease that is mediated by a variety of cells, including keratinocytes, T cells, endothelial cells, macrophages, and dendritic cells [27]. Keratinocytes are a type of resident skin cell that can be both a participant and a victim of psoriasis. The balance between the proliferation and apoptosis of keratinocytes is essential for maintaining skin homeostasis. In psoriatic lesions, skin homeostasis becomes imbalanced. It has been observed that the apoptosis of keratinocytes is reduced, which inevitably leads to hyperproliferation [28,29]. Hyperproliferative keratinocytes produce an excessive amount of cytokines to sustain and amplify the inflammatory response [4,5,6]. Alantolactone has the potential to reduce hyperproliferation and/or the strong  inflammatory response that is a result of keratinocytes hyperproliferation. Therefore, alantolactone is a potential therapeutic candidate for psoriasis.

Alantolactone is a natural sesquiterpene lactone compound isolated from *Inula helenium* and *Radix inulae* with multiple pharmacological activities, including antifungal, anthelmintic, antimicrobial, anti-inflammatory, and antitrypanosomal activities, and antiproliferative effects on several cancer cell lines, including colon, melanoma, ovary, prostate, lung, and leukemia cell lines [9,10,11,12,13,14,15,16]. M5 cytokines were used to simulate keratinocytes, thereby establishing a psoriasis keratinocyte model in vitro [20,22,23]. In the present study, the results showed that M5 cytokines can induce HaCaT keratinocytes to generate some characteristics of psoriatic keratinocytes. Interestingly, alantolactone treatment attenuated the hyperproliferation induced by M5 cytokines (Figure 1A) and partially reduced the expression of KRT6, a hallmark of psoriasis hyperproliferation (Figure 1B). In addition, TNF-α, IL-6, and IL-1β are important proinflammatory cytokinesthat are related to the pathogenesis of psoriasis [30,31]. The IL-8 chemokine promotes the pathogenesis of psoriasis by recruiting neutrophils, monocytes, and T cells to psoriatic lesions [32,33,34,35]. In this study, M5 cytokines increased the proinflammatory expression of TNF-α, IL-6, IL-1β, and IL-8. Whereas, alantolactone was able to significantly decrease these proinflammatory cytokines in the M5 cytokine-stimulated HaCaT keratinocytes (Figure 2). These results indicated that alantolactone could attenuate the excessive inflammatory response of HaCaT keratinocytes induced by M5 cytokines.

It has been reported that the STAT3 protein plays a key role in the occurrence and development of psoriasis [36,37]. NF-κB is a protein transcription factor that plays an important regulatory role in immune and inflammatory pathways, cell apoptosis, and proliferation [38]. A previous study showed that the level of active phosphorylated NF-κB is elevated in psoriasis [39]. Alantolactone could inhibit NF-κB pathway activation, which would subsequently suppress inflammation [14,18]. However, the effect of alantolactone on the STAT3/NF-κB pathway in M5 cytokine-stimulated HaCaT cells has not been previously reported. The results of the study showed that M5 cytokines stimulated the activation of the STAT3/NF-κB pathway, while alantolactone treatment significantly reduced the activation of the STAT3/NF-κB pathway in M5 cytokine-stimulated HaCaT keratinocytes. Moreover, the mechanism of action of alantolactone, by which it suppresses inflammatory keratosis, may also result from the activation of nuclear factor erythroid-2-related factor-2 (Nrf2). It was demonstrated that alantolactone was capable of inducing nrf2 signaling, activating antioxidative genes like heme oxygenase-1 (HO-1) and subsequently, leading to ferroptosis on target cells [18,40]. Nevertheless, the exact mechanism remains unsettled and is worthy of further exploration.

IMQ is a widely accepted method to induce a psoriasis-like state in a mouse model. This model is very similar to human psoriasis with similar histological and clinical features, including erythema, scaling, epidermal hyperplasia, and inflammatory cell infiltration [19]. We further evaluated the therapeutic effects of alantolactone using this IMQ-induced psoriasis-like dermatitis mouse model and measured the immune regulation mechanism. Interestingly, alantolactone treatment significantly ameliorated these symptoms (Figure 4 and Figure 5).

In addition, IMQ-induced psoriatic dermatitis in mice resulted in significantly increased mRNA levels of TNF-α, IL-6, IL-1β, IL-8, IL-17A, and IL-23 in skin lesions of mice (Figure 6). TNF-α is produced by keratinocytes and plays an important role skin inflammation [41]. The IL-17A cytokine promotes the accumulation of inflammatory cell infiltration in the epidermis and dermis, which affects the functionality of the skin barrier [42]. IL-23 cytokine is also overexpressed in psoriatic skin lesions compared with normal skin lesions [43]. A previous study showed that the improvement of psoriasis is caused by the suppression of Th1- and Th17-mediated cytokines, which has been shown in both psoriasis patients and IMQ-induced mice [44]. The present study showed that alantolactone decreased the mRNA levels of TNF-α, IL-6, IL-1β, IL-8, IL-17A, and IL-23, thereby suppressing Th1- and Th17-mediated cytokines and improving IMQ-induced psoriasis-like skin lesions and inflammation status in mice.

The current therapeutic options available for psoriatic patients are mostly topical ointments or creams with steroids or dithranol. Such a topical treatment can efficaciously reduce itching and prevent further cell proliferations. However, the effectiveness is brief, and psoriasis tends to be recurrent and incurable [45]. Systemic administration of antipsoriatic drugs, such as methotrexate or ciclosporin, appears to be an alternative approach, but they all possesses different levels of adverse effects, including nausea, lower hemopoiesis, and liver or kidney damage with long-term usage [46]. Consequentially, the pharmaceutical importance of alantolactone as a newer antipsoriasis drug is accentuated.

## 5. Conclusions

Alantolactone can attenuate the proliferation and inflammation of HaCaT keratinocytes induced by M5 cytokines. Moreover, the current study showed that alantolactone could ameliorate the severity of skin lesions and improve inflammation in an IMQ-induced psoriasis-like mouse model. As epidermal hyperplasia and inflammation are hallmarks of psoriasis, our new findings indicate that alantolactone can not only alleviate these skin lesions by inhibiting inflammation but also relieve epidermal hyperplasia by reducing the expression of TNF-α, IL-6, IL-1β, IL-8, IL-17A, and IL-23. These factors make it an attractive candidate for future development as an antipsoriatic treatment.

## Data Availability

The data that support the findings of this study are available from the corresponding author upon reasonable request.

## Supplementary Materials

The following are available online at https://www.mdpi.com/article/10.3390/life11070616/s1. Table S1: List of primers used for real-time PCR analysis. Supplementary Figure S1 Western blot: protein expression levels of pSTAT3, STAT3, and I-κBα. SupplementaryFigure S2 Western blot: Protein expression levels of pSTAT3, STAT3, and I-κBα.
