# Supplementary material for: Alantolactone Suppresses Proliferation and the Inflammatory Response in Human HaCaT Keratinocytes and Ameliorates Imiquimod-Induced Skin Lesions in a Psoriasis-Like Mouse Model
Source: Life (Basel). 2021 Jun 25;11(7):616. doi: 10.3390/life11070616 (PMC8303865; doi:10.3390/life11070616)
Supplement: Supplementary file 1 [file life-11-00616-s001.zip › life-1206593-supplementary/life-1206593 supplementary.pdf]

Supplementary materials:

Article

Alantolactone Suppresses Proliferation and the Inflammatory Response in Human HaCaT Keratinocytes and Ameliorates Imiquimod-Induced Skin Lesions in a Psoriasis-Like Mouse Model

Wen-Ho Chuo [1], Yu-Tang Tung [2], Chao-Liang Wu [3], Nicole Bracci [4], Yu-Kang Chang [5,6], Hung-Yi Huang [7*] and Chi-Chien Lin [8,9,10,11*]

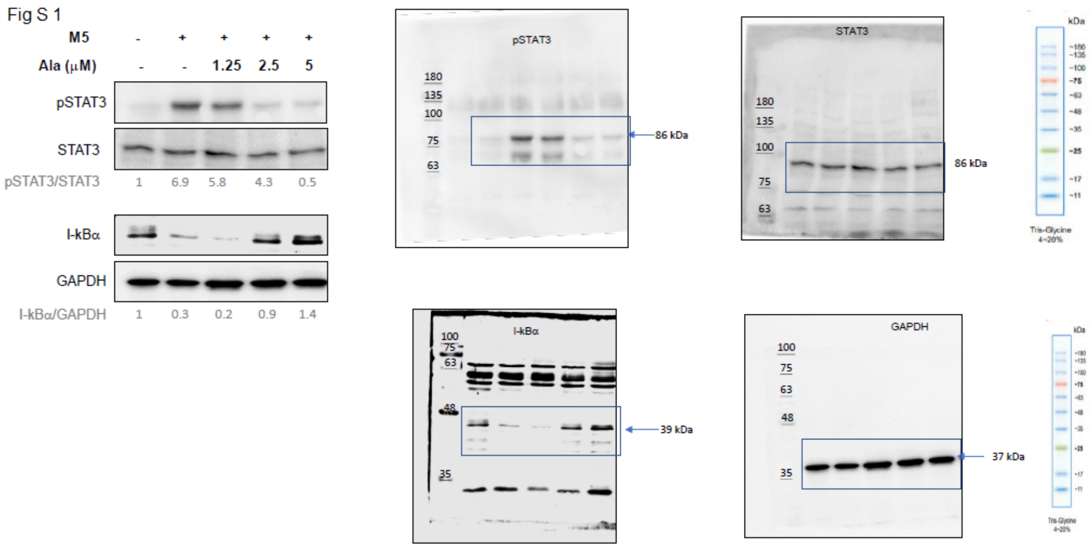

Figure S1. Western blot: protein expression levels of pSTAT3, STAT3, and I-κBα.

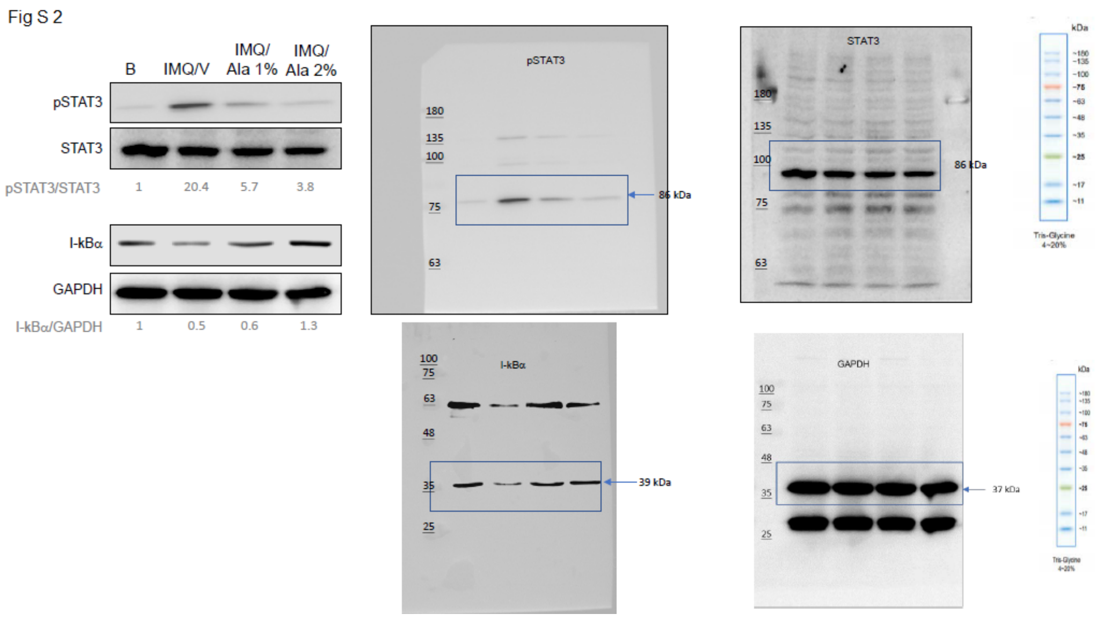

Figure S2. Western blot: Protein expression levels of pSTAT3, STAT3, and I-κBα.
