# Supplementary material for: Alantolactone Suppresses Proliferation and the Inflammatory Response in Human HaCaT Keratinocytes and Ameliorates Imiquimod-Induced Skin Lesions in a Psoriasis-Like Mouse Model
Source: Life (Basel). 2021 Jun 25;11(7):616. doi: 10.3390/life11070616 (PMC8303865; doi:10.3390/life11070616)
Supplement: Supplementary file 1 [file life-11-00616-s001.zip › life-1206593-supplementary/Suplemetary life-1206593.pdf]

Supplementary Table S1. List of primers used for real-time PCR analysis

Gene	Forward primer	Reverse primer
KRT6	5'- TCTGGACCTGGACAGCATCA-3'	5'- CTCTTTAGGGCAATCTCCTCGTACT -3'
TNF-α	5'-TGCTCCTCACCCACACCAT-3'	5'-GGAGGTTGACCTTGGTCTGGTA-3'
IL-6	5'-CCCACACAGACAGCCACTCA-3'	5'-TCGAGGATGTACCGAATTTGTTT-3'
IL-1β	5'-ACGAATCTCCGACCACCACTA-3'	5'-TCCATGGCCACAACAACTGA-3'
IL-8	5'-TGATTTCTGCAGCTCTGTGTGA-3'	5'-GGGTGGAAAGGTTGGAGTAGTATG-3'
IL-17A	5'-CCCAAAAGGTCCTCAGATTACATCA-3'	5'-TCATTGCGGTGGAGATTCC-3'
IL-23	5'-CCTTCTCCGCTTCAAAATCCTT-3'	5'-ACCCGGGCGGCTACA-3'
GAPDH	5'-ACCCAGAAGACTGTGGATGG-3'	5'-TGCTGTAGCCAAATTCGTTG-3'
